# Absence of Membrane Phosphatidylcholine Does Not Affect Virulence and Stress Tolerance Phenotypes in the Opportunistic Pathogen *Pseudomonas aeruginosa*

**DOI:** 10.1371/journal.pone.0030829

**Published:** 2012-02-17

**Authors:** Adel A. Malek, Matthew J. Wargo, Deborah A. Hogan

**Affiliations:** 1 Department of Microbiology and Immunology, Dartmouth Medical School, Hanover, New Hampshire, United States of America; 2 Department of Microbiology and Molecular Genetics, University of Vermont College of Medicine, Burlington, Vermont, United States of America; Institut de Pharmacologie et de Biologie Structurale, France

## Abstract

During growth in presence of choline, both laboratory and clinical *Pseudomonas aeruginosa* strains synthesize phosphatidylcholine (PC), and PC makes up ∼4% of the total membrane phospholipid content. In all the strains tested, PC synthesis occurred only when choline is provided exogenously. Mutants defective in synthesis of PC were generated in the strain backgrounds PAO1 and PA14. Minimum inhibitory concentration studies testing sensitivity of PC-deficient strains towards various antibiotics and cationic antimicrobial peptides revealed no differences as compared to wild-type strains. Mutants incapable of synthesizing PC were also found to be unaffected in motility and biofilm formation on abiotic surfaces, colonization of biotic surfaces and virulence in a mouse infection model. A global phenotypic microarray was further used to identify conditions wherein membrane PC may play a role of in *P. aeruginosa*. No culture conditions were identified wherein wild-type and PC-deficient mutants showed phenotypic differences. Membrane PC may serve a highly specific role during *P. aeruginosa* interactions with its eukaryotic hosts based on all the clinical strains tested retaining the ability to synthesize it during availability of choline.

## Introduction

Phosphatidylcholine (PC) is an essential phospholipid in eukaryotes, where it is a critical structural component of cell membrane, and plays key roles in signaling pathways [1], [2], [3]. In contrast, only 10% of prokaryotes synthesize PC, with a higher frequency in bacterial symbionts and pathogens [4], [5]. In bacteria that produce PC, synthesis occurs mainly using two pathways: the Pmt- and Pcs-dependent pathways. The Pmt pathway, which is also conserved in eukaryotes, allows synthesis of PC de novo [6]. This pathway involves sequential methylation of phosphatidylethanolamine by phospholipid *N*-methyltransferases (PmtA) to yield phosphatidylcholine. The more recently discovered Pcs pathway is unique to bacteria [7]. In this pathway, the enzyme phosphatidylcholine synthase (Pcs) catalyzes the condensation of choline directly with CDP-diacylglycerol to form PC.

Several studies have shown that bacterial membrane PC can be important in host-associated bacteria for symbiosis or pathogenesis. PC-deficient mutants of *Bradyrhizobium japonicum* and *Sinorhizobium meliloti* exhibit drastically reduced symbiosis with their plant hosts [8], [9]. The plant pathogen *Agrobacterium tumifaciens* requires PC for assembly of the T4SS components which are critical for formation of crown-gall tumors on plants [10], [11]. In the human pathogen *Legionella pneumophila*, PC-deficient mutants were attenuated for virulence and had increased susceptibility to macrophage-mediated killing [12]. These defects were attributed to decreased effector translocation by the Dot/ICM T4SS, poor adhesion to macrophages and decreased steady state levels of flagellin [12]. In *Brucella abortus*, the *pcs* mutant had an altered cell envelope and was unable to establish a replication niche inside the macrophages, and showed a severe virulence defect in a mouse model of infection [13], [14].

*Pseudomonas aeruginosa* synthesizes the phospholipids phosphatidylethanolamine, cardiolipin, and phosphatidylglycerol and alanyl-phosphatiydylglycerol de novo. A report by Wilderman et al. [15] has shown that *P. aeruginosa* can also synthesize PC. It was further shown that PC production in *P. aeruginosa* occurs exclusively in the presence of choline and that synthesis is dependent on Pcs [15]. However, the significance of PC in *P. aeruginosa* membranes for commonly assayed phenotypes had not been investigated.

In this study, we used a variety of assays for assess the phenotypes of the *pcs* mutant relative to the wild-type *P. aeruginosa*. We focused on testing whether membrane PC formation impacts antibiotic resistance, biofilm formation and virulence which are critical aspects of *P. aeruginosa* physiology in vivo during infections. To take an unbiased approach for the characterization of *pcs* mutants, we also used a Biolog global phenotypic microarray to identify culture conditions wherein membrane PC may play a role of in *P. aeruginosa*. Either approach revealed no phenotypic differences between wild-type or PC-deficient mutants suggesting that membrane PC is dispensable for stress tolerance and virulence-related attributes in *Pseudomonas aeruginosa*.

## Results and Discussion

### Laboratory and clinical strains of *P. aeruginosa* produce PC in a choline dependent manner

**Figure 1:**
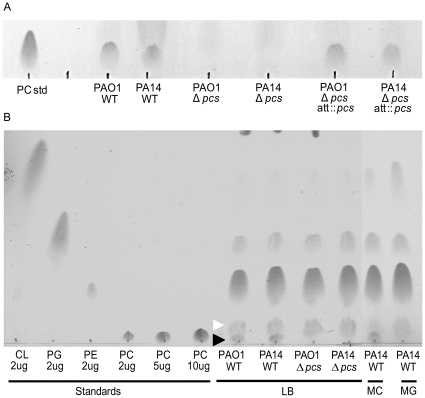
Phospholipid profiles of *P. aeruginosa* strains grown in rich and minimal media. Separation of phospholipids by 1-D thin layer chromatography and detection by charring with sulphuric acid-methanol solution (1∶19, v/v) is shown. (A) Panel showing complementation of the PAO1 and PA14 Δ*pcs* deletion mutants with *pcs* gene expressed in trans at a separate genomic location (*att*::*pcs*). Phospholipids were extracted from PAO1 and PA14 strains grown overnight in MOPS-20mM choline. (B) Phospholipid profiles of PAO1 and PA14 strains grown overnight in LB, MOPS-20mM glucose and MOPS-20mM choline. TLC spots corresponding to PC are shown as black arrowheads. The nature of the spot corresponding to white arrowheads is observed under all growth conditions just above the PC migration front remains unknown. This spot has been observed under all growth conditions, regardless of the presence of choline in the medium. Panels A and B are representative images of observations from three independent experiments. (Slight differences in the migration fronts of PL standards and the extracted phospholipids are likely a result of their acyl chains varying in lengths and saturation levels).

To study the role of PC in *P. aeruginosa* membranes, mutants with in-frame deletions in the phosphatidylcholine synthase (*pcs*) gene were constructed in *P. aeruginosa* PAO1 and PA14, two laboratory strains. The wild types and their mutant derivatives were grown in a defined medium with choline and the profiles of extracted phospholipids were analyzed by thin layer chromatography. The PAO1 and PA14 Δ*pcs* mutants completely lacked PC, and these defects were complemented by expression of the *pcs* gene from a separate genomic location (Fig. 1A). PC is produced by both PAO1 and PA14 strains when grown in LB and MOPS medium supplemented with choline medium but not in MOPS medium without choline (Fig. 1B). In LB grown *P. aeruginosa*, PC constitutes to 4% of the total phospholipids, while phosphatidylethanolamine, phosphatidylglycerol and cardiolipin contribute to 73%, 17% and 5% respectively of the total phospholipids [16]. Similar profiles were observed in wild-type strains grown in MOPS medium with choline (Fig. 1B). Our results were consistent with those from a previous study in PAO1 [15], and show that PC synthesis in *P. aeruginosa* occurs exclusively during availability of choline, and requires the activity of Pcs.

Several studies have shown that during chronic infections, *P. aeruginosa* acquires pathoadaptive mutations that confer benefits resulting in enhanced persistence and colonization within the host. Some of the characterized mutations include those in genes encoding regulators of alginate biosynthesis [17], motility components [18], regulators of quorum sensing [19] and T3SS [20]. Therefore, we tested clinical strains of *P. aeruginosa* isolated from sputum of patients chronically infected with *P. aeruginosa* due to cystic fibrosis to identify whether they have the ability to synthesize PC and whether the relative amounts were similar. Clinical strains DH # 220, 228, 214 and 217 were tested, and it was found that these strains synthesized PC following growth in LB, at amounts similar to that produced by the PAO1 strain (Fig. S1). Seven additional clinical isolates were then tested for synthesis of PC in LB as well as MOPS media with or without choline. As shown in Table S1, all of the strains tested synthesized PC after growth either in LB or MOPS medium with choline. However, none of the strains produced PC after growth in minimal medium without choline. These data show that *P. aeruginosa* clinical strains have the ability to produce PC, and that PC production requires choline, and this is likely dependent on the activity of Pcs as in WT strains.

### PC-deficient mutants are not affected in their sensitivity towards antibiotics and antimicrobial peptides

Liposome studies have shown that artificially generated PC-rich membranes are relatively more resistant to cationic antimicrobial peptides (CAMPs) [21]. Additionally, in *Haemophilus influenzae*, decoration of LPS with phosphorylcholine epitopes, results in increased resistance to the human cationic peptide LL-37, presumably due the orientation of the positively charged amine group of choline towards the outer surface of bacteria, effectively decreasing the binding affinity of CAMPs [22]. Based on these studies, we hypothesized that PC production in *P. aeruginosa* could specifically alter resistance to cationic antimicrobial peptides. To test this, the sensitivity of PAO1 Δ*pcs* mutant to the CAMPs: polymyxin-B and human LL-37 was assayed. Table 1 shows that no differences in MICs towards these peptides were observed between the Δ*pcs* mutant and WT. Thus loss of PC in *P. aeruginosa* does not alter resistance to CAMPs.

**Table 1:** A PC-deficient *P. aeruginosa* mutant resembles WT in its sensitivity to antibiotics and antimicrobial peptides.

	MIC µg/ml		
	Growth medium and strains		
	MOPS-20mM Glucose	MOPS-20mM Choline	
Antibiotics and antimicrobial peptides	PAO1 WT	PAO1 WT	PAO1 Δ*pcs*
**Ciprofloxacin**	2	2	2
**Tobramycin**	4	4	4
**Gentamicin**	1.125	1.125	1.125
**Kanamycin**	125	125	125
**Tetracycline**	75	75	75
**Polymyxin**	2.25	2.25	2.25
**LL-37**	3	3	3

MICs (µg/ml) were determined by serial two-fold dilution method.

In *Brucella abortus*, *a* PC-deficient mutant was proposed to have altered cell surface properties compared to the WT strain, based on changes in its resistance profiles to a broad range of antibiotics [14]. To test if PC that is synthesized when choline is available affected antibiotic resistance, we tested the sensitivity of PAO1 Δ*pcs* mutant to a variety of antibiotics and compared it to PAO1 WT. In *P. aeruginosa*, the ability to synthesize PC was not found to affect the sensitivity towards any of the antibiotics tested (Table 1), suggesting that the properties of membranes are not altered drastically in PC-deficient strains.

In comparison to *B. abortus*, where PC is a major component of the phospholipid membranes (28% of the total phospholipid) [13], PC is a minor component in *P. aeruginosa* membranes (4% of the total phospholipid) (Fig. 1B). Based on the lack of phenotype of Δ*pcs* mutant in MIC experiments, it is possible that the relatively small amount of PC produced by *P. aeruginosa*, in choline containing media, does not alter the effective surface charge of the membranes. To test this, we compared the binding of Cytochrome C to PAO1 WT and PAO1 Δ*pcs* mutant. Cytochrome C is highly positively charged and the binding of the molecule is dependent on the negative charge of the bacterial surface [23]. No differences were observed between the PAO1 WT and PAO1 Δ*pcs* mutant in the Cytochrome C binding assay (data not shown), suggesting that synthesis of PC by the activity of Pcs does not grossly alter the overall cell surface charge of *P. aeruginosa*.

### PC-deficient mutants are not altered in their motility and biofilm formation on abiotic surfaces

**Figure 2:**
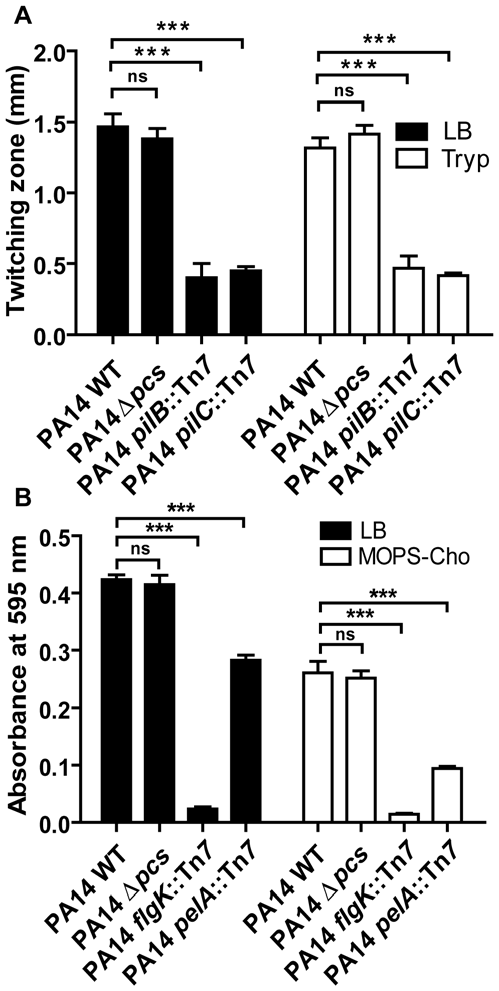
PC-deficient mutants are not affected for motility and biofilm formation on abiotic surfaces. (A) Twitching motility assays of PA14 strains on LB and tryptone agar plates were performed as previously described [28]. Motility was determined by measuring diameter (mm) of the zone of expansion from the point of inoculation. (B) Biofilm assays of PA14 strains grown in LB and MOPS-20mM choline media were performed using 96-well PVC microtiter plates as previously described [28]. At 12 hours, the biofilms formed were stained with crystal violet, solubilized with 95% ethanol, and quantified by measuring absorbance at 595 nm. In panels A and B, values represent averages and error bars are SD (n = 3). Tests of significance were conducted using a one- way analysis of variance (ANOVA) with a Bonferroni multiple comparison test, ***P<0.001, ^ns^ *P*>0.05.

*P. aeruginosa* displays three types of motility: type IV pilus-mediated twitching motility and flagellum-mediated swimming and swarming motilities. All these forms of motility involve complex membrane-spanning systems, in the form of flagellar or pili components [24]. Based on this, we hypothesized that changes in *P. aeruginosa* membranes due to PC production may impact motility. Fig. 2A shows the results of a twitching motility assay performed on tryptone and LB agar plates (membrane PC is produced under these medium conditions, data not shown). While the pili-defective mutants had 4- and 3.5-fold smaller diameters of twitching zones compared to PA14 WT on LB and tryptone plates respectively (P values<0.001), the zones of twitching in PC deficient mutants resembled that of WT. Similarly, standard motility plate-based assays showed that PC deficient strains were not affected in swimming and swarming motilities (data not shown).

*P. aeruginosa* also forms biofilms on abiotic surfaces that are highly complex and differentiated, and defects in motility alter biofilm formation [25]. The capacity of *P. aeruginosa* to form biofilms is thought to be an important for its ability to cause chronic infections and for persisting on implanted medical devices [26]. The PC-deficient strains were assayed for biofilm formation on plastic using 96-well microtiter plates. While the *pel* mutant, which is defective in synthesis of extracellular matrix [27], and the flagellum-deficient *flgK* mutant [28] formed biofilms that were significantly smaller than WT (P values<0.001) as previously published, PC-deficient mutants were not altered in their ability to form biofilms (Fig. 2B). These data show that PC formation does not impact *P. aeruginosa* motility or biofilm formation on abiotic surfaces.

### PC is not required for colonization of biotic surfaces or virulence

To assess the role of PC in host cell-pathogen interactions, PC-deficient *P. aeruginosa* strains were tested for colonization of eukaryotic host surfaces using two model systems: the human airway epithelial cell co-culture model [29], [30] and the fungal co–culture model [31].

**Figure 3:**
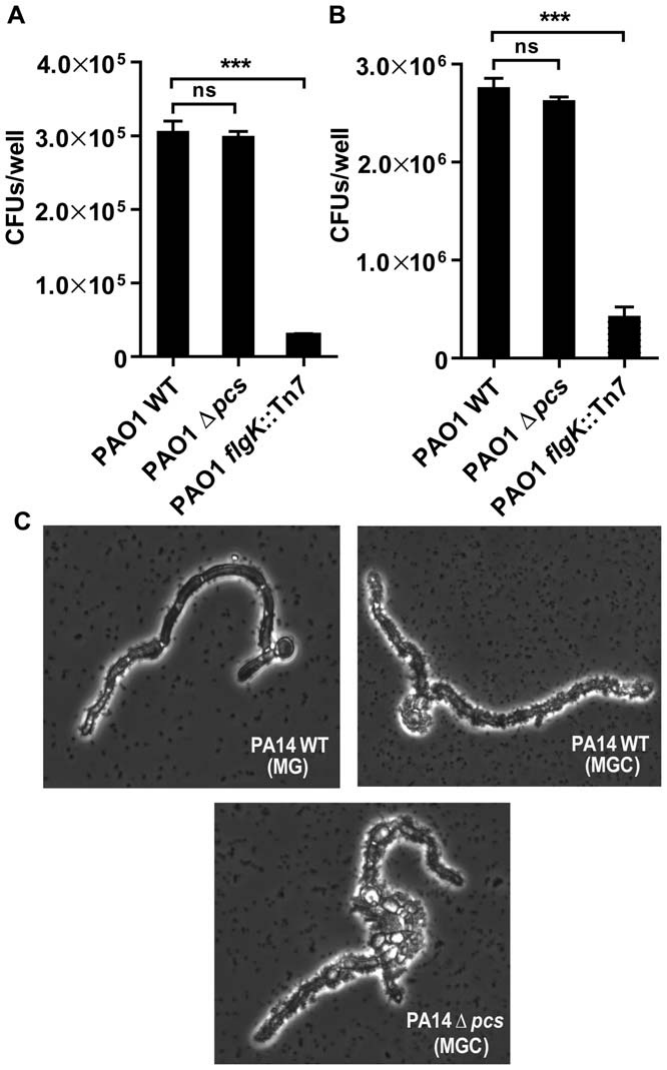
PC-deficient mutants are not affected for colonization of biotic surfaces. Initial attachment and biofilm formation of PAO1 strains on epithelial cells was performed as previously described [29], [30]. Seven-day-old confluent monolayers of CFBE epithelial cells were inoculated with bacterial strains and CFUs were estimated at (A) 1 hour post infection for enumerating initial attachment and (B) 6 hours post infection for enumerating biofilm formation. Values in A and B represent averages and SD from three independent replicates, and statistical significance of data was evaluated using a two-tailed unpaired *t* test, ***P<0.001, ^ns^ *P*>0.05. (C) Phase contrast micrographs show 48-hour biofilms formed by *P. aeruginosa* PA14 strains on the fungus *Candida albicans*. PA14 strains were co-cultured with constitutively-filamentous *Candida albicans nrg1/nrg1* strain as previously described [31]. The media used were MG (MOPS 30 mM glucose) and MGC (MOPS 20 mM glucose, 10 mM choline). Note: Presence of choline enhances colonization. The micrographs are representative images of from three independent experiments.

In the airway epithelial cell model, *P. aeruginosa* PAO1 colonization was assessed after co-culture for 1 hour for enumerating initial attachment and after 6 hours for enumerating biofilm formation. At each of these time points, microscopic examination revealed that the integrity of epithelial cell monolayers had not been compromised following addition of *P. aeruginosa* (data not shown). Fig. 3A shows that after 1 h post inoculation, about 25% of the cells present in the initial inocula of *P. aeruginosa* PAO1 WT and PAO1 Δ*pcs* had attached to the epithelial cells. In contrast, the PAO1 *flgk*::Tn*7* mutant showed a 15-fold lower initial attachment (P value <0.001), as has been described previously [30]. At 5 hours post initial attachment, a 10-fold increase in both *P. aeruginosa* PAO1 WT and PAO1Δ*pcs* CFUs was observed due to growth of the strains, whereas the PAO1 *flgk*::Tn*7* showed 5.5 fold lower CFUs (P value <0.001) compared to the PAO1 WT and PAO1Δ*pcs* strains (Fig. 3B).

In the fungal co-culture model, PA14 strains and the constitutively-filamentous *Candida albicans nrg1/nrg1* strains were cultured for 48 hours. Under these conditions *P. aeruginosa* PA14 WT attach, form biofilms on the fungal surface, and eventually kill the fungus [31]. As shown in Fig. 3C, the addition of choline to the culture medium enhanced PA14 WT biofilm formation. However, comparison of PA14 WT and PA14Δ*pcs* biofilms on the fungal surface in medium with choline revealed no distinguishable differences (Fig. 3C).

**Figure 4:**
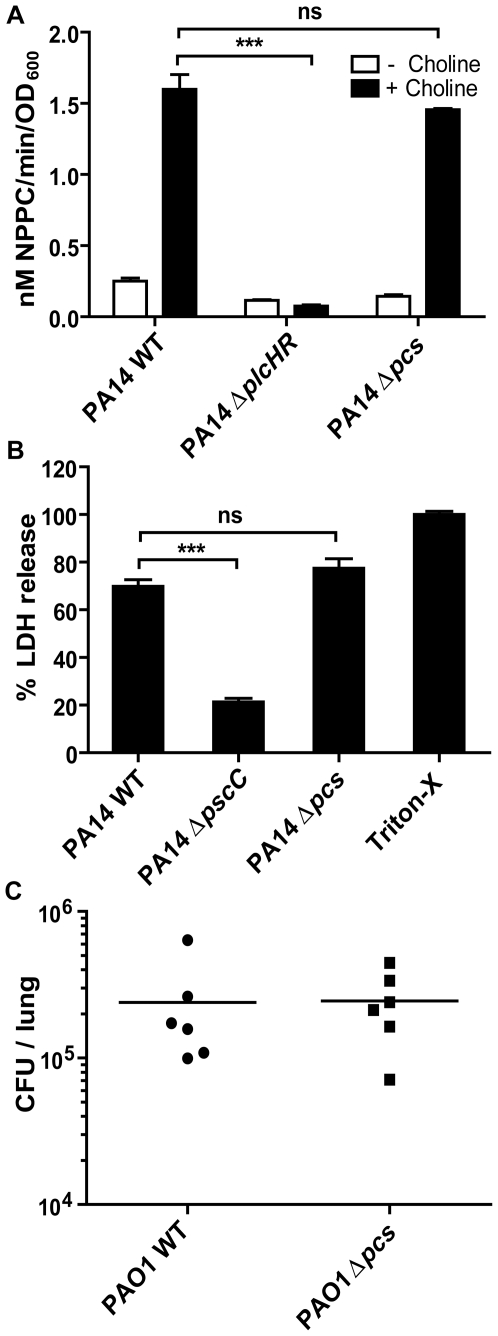
PC-deficient mutants are not defective in virulence. (A) PA14 PlcH activity was assayed for by measuring NPPC hydrolysis activity in defined medium with (black bars) or without (white bars) choline. (B) PA14 T3SS-mediated cytotoxicity towards epithelial cells was assessed by monitoring LDH release 6 hours post infection. LDH released from control cells lysed with Triton-X 100 was set as 100% cytotoxicity. In A and B, error bars represent SD of the means of triplicate experiments (C) Bacterial burden 24 hours post infection from whole homogenized lungs of mice inoculated at a high infective dose. CFU counts from individual mice are plotted with means of each group denoted by horizontal lines. Means were not significantly different. Statistical significance of data in panels A, B and C were evaluated using a two-tailed unpaired *t* test, ***P<0.001, ^ns^ *P*>0.05.

Several studies have shown hemolytic phospholipase C (PlcH) and T3SS to play key roles in *P. aeruginosa* virulence towards its eukaryotic hosts [31], [32], [33], [34], [35], [36], [37], [38], [39]. Using in vitro assays, we tested whether changes in *P. aeruginosa* membranes due to PC synthesis affects the production of these virulence factors. PlcH activity was analyzed using the artificial substrate p-nitrophenyl-phosphorylcholine (NPPC) [40]. Cultures of PA14 WT, Δ*pcs* and Δp*lcHR* were grown in defined medium with or without choline and the supernatants were analyzed for NPPC activity. In WT supernatants, presence of choline in the medium resulted in 6-fold higher phospholipase C activity while no increase in activity was observed in the Δ*plcHR* strain, a mutant defective in synthesis of phospholipase C (P value <0.001). In comparison, no difference in induction of NPPC activity was observed in Δ*pcs* mutant as compared to WT (P value >0.05) (Fig. 4A). While *P. aeruginosa* PAO1 strains can form biofilms on airway epithelial cells in a co-culture model as described above, PA14 strains are highly cytotoxic and lyse the epithelial cells, in a T3SS-dependent manner [29], [30]. T3SS-mediated virulence was assayed in PA14 strains by analyzing cytotoxicity towards airway epithelial cells. A PA14 Δ*pscC*, strain, with a mutation in a structural component of the T3SS translocase, showed 4-fold lower percent cytotoxicity as compared to PA14 WT (P value <0.001). In comparison, PA14 Δ*pcs* was not defective in T3SS mediated-cytotoxicity towards epithelial cells (Fig. 4B).

To assess the role of membrane PC in fitness in vivo during infections, we tested PAO1 WT and the isogenic *pcs* mutant in a mouse model of acute pneumoniae [41]. Following oropharyngeal inoculation of equivalent ratios of PAO1 WT and PAO1 Δ*pcs* mutant into C57Bl/6J mice (3×10^7^ CFU/animal), numbers of CFUs recovered from the lungs were determined at 24 hours post infection. No differences in bacterial burden were observed 24 hours post infection suggesting that the absence of PC did not affect virulence in vivo (Fig. 4C). In addition, levels of infiltrating white blood cells were similar in PAO1 WT and PAO1Δ*pcs* mutant-infected mice (Fig. S2), suggesting that there were no significant differences in airway inflammation.

These data collectively suggest that absence of PC in *P. aeruginosa* membranes does not affect colonization of biotic surfaces, virulence and the ability to cause an efficient infection process in mice.

### Comparison of PA14 WT and PA14 Δpcs using Biolog phenotypic microarrays

Our attempts to identify a role of PC, using a candidate approach focused at aspects of *P. aeruginosa* persistence, virulence and antibiotic stress survival could not detect any obvious associated phenotypes in the PC-deficient strains. Based on this, we took an unbiased approach and compared a PC-deficient mutant and WT using a Biolog phenotypic microarray (Biolog, Hayward, CA), to identify the role of membrane PC in *P. aeruginosa* physiology. The assays were performed with IF-10 medium (Biolog, Hayward, CA) supplemented with 1 mM choline chloride. Under these conditions, *P. aeruginosa* formed PC in its membranes as determined by analysis of phospholipid profiles by TLC (data not shown). In this study, PM plates 9–20 were used which allowed analysis of growth under 1152 different culture conditions (http://www.biolog.com/pdf/PM11-PM20.pdf). This was used for a comparative analysis of sensitivity towards variations in osmolarity and pH, and a suite of antibiotics, antimicrobial peptides and chemical inhibitors. A similar strategy has previously been used to assign a functional role to the phospholipid alanyl-phosphatidylglycerol which is formed by *P. aeruginosa* under acidic conditions (pH 5.3), and contributes to 6% to the overall lipid content [42]. The study found alanyl-PG to confer resistance to killing by cationic peptide protamine, the osmolyte sodium lactate and antibiotic cefsulodin.

In 98% of the culture conditions tested in our study, PA14 WT and PA14 Δ*pcs* mutant were indistinguishable in duplicate runs (yellow growth kinetic curves, Fig. S3). No differences were observed in the growth kinetics of PA14 WT and Δ*pcs* mutants in the presence of antibiotics: tetracycline (PM12, wells A5–A8), carbenicillin (PM12, wells A9–A12), polymyxin B (PM12, wells B9–B12), tobramycin (PM12, wells F1–F4), ciprofloxacin (PM 20, wells D5–G8), gentamicin (PM11,wells G5–G8) and kanamycin (PM11, wells H5–H8) and the cationic antimicrobial peptide: protamine sulphate (PM16, wells C5–C8) (Fig. S3) which is consistent with our previous observations in MIC experiments and our hypothesis that the absence of PC does not drastically alter the physicochemical properties or the overall surface charge of *P. aeruginosa* membranes (Table 1). Although a few differences were observed (listed in Fig. S3), none of these differences were present in both replicate runs. Two of the compounds tested that showed differences in their effects on the *pcs* mutant as compared to the WT in one of the two replicates, sodium lactate and 5-fluoroorotic acid, were examined more closely. Growth curves studies were performed in the presence of these compounds. No differences were observed in the growth rates of PA14 Δ*pcs* when compared with PA14 WT strain (data not shown). Based on these results, no conditions could be identified wherein PA14 WT and PA14 Δ*pcs* mutants showed phenotypic differences.

### Concluding Remarks

Comparative analysis of the various sequenced non-pathogenic and pathogenic Pseudomonads revealed the *pcs* gene to be present exclusively in the latter. The analysis showed that while *P. aeruginosa*, the plant pathogens *P. syringae* and P. *fluorescens*, and the entomopathogenic *P. entomophila* harbor the *pcs* gene, the free living environmental strains *P. stutzeri* and *P. putida* do not have the *pcs* gene. Based on these observations, we had hypothesized that the synthesis of PC could be important for *P. aeruginosa* host-pathogen interactions or could confer benefits by promoting survival under stressful conditions. Our study shows that *P. aeruginosa* PC-deficient mutants are indistinguishable from WT in motility and biofilm formation on abiotic surfaces, colonization of biotic surfaces, and virulence. Furthermore, MIC assays and Biolog phenotypic microarray experiments suggest that in the absence of PC, the properties of the membranes and overall surface charge of the bacteria are not drastically affected. Since PC is produced in relatively small amounts, it is possible that its absence is either tolerated or compensated by a slight increase in synthesis of the zwitterionic phospholipid phosphatidylethanolamine, which is a major phospholipid in *P. aeruginosa* membranes (Fig. 1, 2). However, the fact that all of the clinical strains of *P. aeruginosa* tested had retained the ability to synthesize PC suggests that it might serve a highly specialized function during interaction with eukaryotic hosts. It has been reported that phospholipid environment can affect topological organization and assembly of membrane proteins [43], [44]. Based on this, one can imagine that PC could aid in assembly or localization of specific proteins in *P. aeruginosa*. This can be investigated in the future by using a comparative proteomics approach analyzing the membrane proteome profiles of WT and PC-deficient strains of *P. aeruginosa*.

## Materials and Methods

### Ethics statement

The protocol for animal infection was approved by the University of Vermont Institutional Animal Care and Use Committee, in accordance with Association for Assessment and Accreditation of Laboratory Animal Care guidelines. All procedures were under pentobarbital anesthesia and all efforts were made to minimize animal suffering.

### Bacterial strains, plasmids and growth conditions

The bacterial strains and plasmids used in this study are listed in Table 2. *P. aeruginosa* and *E. coli* strains were maintained on LB medium at 37°C. LB, a rich medium, and morpholinepropanesulphonic acid (MOPS) medium [45], a defined medium were used for growing the bacteria for the various assays. Glucose or choline was added at concentrations described in the text as carbon sources in MOPS medium. When necessary, antibiotics were used at the following concentrations (µg ml^−1^): gentamicin (75) and kanamycin (500) for *P. aeruginosa*, and gentamicin (15) and carbenicillin (75) for *E. coli*. All liquid cultures were grown at 37°C with vigorous aeration.

**Table 2:** Strains and primers used in this study.

Strain/plasmid	Description	Lab reference #	Source or reference
***P. aeruginosa*** ** strains**			
PA14 WT	*P. aeruginosa* PA14 wild type	DH122	[53]
PA14 *Δpcs*	In-frame deletion mutant of *pcs*	DH606	This study
PA14 *Δpcs att::pcs*	*Δpcs* complemented at *att* site	DH1372	This study
PA14 *flgK*::Tn*7*	Tn*7* insertion in *flgK*	DH2	[28]
PA14 *pilB*::Tn*7*	Tn*7* insertion in *pilB*	DH11	[28]
PA14 *pilC*::Tn*7*	Tn*7* insertion in *pilC*	DH12	[28]
PA14 *pelA*::Tn*7*	Tn*7* insertion in *pelA*	DH97	[27]
PAO1 *ΔpscC*	In-frame deletion mutant of *pscC*	DH95	This study
PAO1 WT	*P. aeruginosa* PAO1 wild-type	DH395	[54]
PAO1 *Δpcs*	In-frame deletion mutant of *pcs*	DH909	This study
PAO1*Δpcs att::pcs*	*Δpcs* complemented at *att* site	DH920	This study
PAO1 *flgK*::Tn*7*	Tn*7* insertion in *flgK*	DH1072	[28]
PAO1 *ΔplcH*	In-frame deletion mutant of *plcH*	DH860	[55]
**Plasmids**			
pEX18Gm	Integrating vector in *P. aeruginosa* for generating in-frame deletions, Gm^r^		[56]
pUC18miniTn7TGm	Integrating vector in *P. aeruginosa* for generating gene insertions at *att*::Tn7 sites		[47]
**Primers**			
*pcs* in-frame deletion			
GOI-F1	GGTTCTCCTACGCCGACGCCAA		
SOE-GOI-R1	TCGCGCCGGGTCGTTCGATAGGTTCACGGG		
SOE-GOI-F1	CCCGTGAACCTACGAACGACCCGGCGCGA		
GOI-R1	CAGCGAGCCGAAGAACACCGGCT		
*pcs* complementation at *att* sites			
*pcs*R-Fw	AGCACCGACAATCCGATAAC		
*pcs*R-Rev	TCCTTGCGATGATAGGGCT		

### Construction of *P. aeruginosa* PAO1 and PA14 Δ*pcs* mutant strains

In-frame deletion constructs for *pcs* were generated using splice-overlap-extension PCR to amplify and splice the 1-kb regions immediately upstream and downstream of the *pcs* gene. The primers (Table 2) were designed to delete the regions between the first and last ten amino acids of the *pcs* ORF. The splice overlap extension PCR products were subcloned into the pCR 2.1 TOPO (Invitrogen, USA) and sequenced. The insert was then cut and ligated into the pEX18-Gm vector and the deletion mutations in *P. aeruginosa* were obtained by recombination, as described previously [46]. Briefly, the pEX18-Gm suicide vector containing the deletion construct was transformed into *E. coli* strain S17/λpir. This *E. coli* strain was then mated with the recipient *P. aeruginosa* strain and single crossover mutants were selected for growth on gentamicin. Recombinants were screened for loss of the *pcs* gene by PCR, after selecting for double crossover events and concomitant loss of *sacB* genes by growth on 5% sucrose LB plates with no NaCl.

### Complementation of the *P. aeruginosa* Δ*pcs* strains

To generate the *att::pcs* complementation construct, the *pcs* gene with 500 bp upstream region was amplified from PAO1 and PA14 genomic DNA using the primers described in Table 2. Each PCR product was subcloned into the pCR 2.1 TOPO (Invitrogen, USA) and sequenced. The inserts were excised and cloned into the pUC18miniTn7TGm plasmid [47]. *P. aeruginosa* strains were co-electroporated with pUC18miniTn7TGm constructs and helper plasmid pTNS2, and integrants were recovered by selection on antibiotic plates. Correct insertion at the *att*::Tn7 site was verified by PCR using primers described by Schweizer et al. [47].

### Extraction and analysis of phospholipids by thin-layer chromatography

Overnight cultures (25 ml) of bacteria grown in rich or minimal medium were centrifuged, and the pellets were washed with water, then weighed. Phospholipids were extracted from the bacterial pellets using a modification of the Folch and Stanley method [48]. Chloroform∶methanol (2∶1 vol/vol) was added at a ratio of 20 ml/g of bacterial pellet. After addition of solvent, the samples were vortexed briefly, agitated at room temperature, and filtered using Whatman filter paper drenched with methanol. 0.2 volumes of 0.9% NaCl was added to filtrates, followed by vortexing, and centrifugation to separate the organic and aqueous phase. After aspiration of the upper phase, the lower phase was dried under nitrogen and dissolved in 100 µl chloroform and stored at −80°C. Total phospholipids in each sample were quantitated using a colorimetric assay, based on phosphorous binding to ammonium ferrothiocyanate [49]. The amount of total phospholipid in the extracted samples was estimated from a standard curve generated using phospholipid standards (range, 0–500 µg). For preparing the phospholipid standards, egg phosphatidylethanolamine and soybean phosphatidylcholine were obtained from Sigma-Aldrich, USA and heart cardiolipin and egg phosphatidylglycerol were obtained from Avanti Polar Lipids, USA. After quantitation, the phospholipids were loaded onto thin Silica Gel 60A plates (Merck, Germany) and separated using a chloroform-methanol-acetic acid (13∶3∶1 vol/vol/vol) solvent system. After the desired solvent front was achieved, the plates were dried and sprayed with sulfuric acid∶methanol (1∶19 vol/vol) solution. The plates were dried again and baked in a high temperature oven till the individual spots could be visualized.

### Antibiotic and antimicrobial peptide minimum inhibitory concentration assays

Minimum inhibitory concentration assays (MICs) for testing sensitivity to antibiotics and antimicrobial peptides were performed using serial two-fold dilution method. The MICs were determined in sterile 96-well flat-bottomed polystyrene microtiter plates (Corning, USA). A (50 ul) two-fold dilution series for each compound was prepared in the microtiter dishes. Bacterial strains to be tested were grown overnight in MOPS medium as described, subcultured and grown to an OD_600_ of 0.4. The ODs of the cultures were adjusted to 0.1, and 50 µl of the cultures were used to inoculate the wells. Wells without any antibiotics served as negative controls and wells without added bacteria and antibiotics served as controls for contamination. The plates were incubated at 37°C for 24 hours. The MICs were defined as lowest concentrations of antibiotics and peptides inhibiting visible growth. All the antibiotic stocks were freshly prepared prior to the assay. Gentamicin, tobramycin, carbenicillin were obtained from Sigma Aldrich, USA; polymyxin B, tetracycline, kanamycin and ciprofloxacin were obtained from RPI, USA; and the human antimicrobial peptide LL-37 was obtained from QCB, USA.

### Motility assays and biofilm formation on abiotic surfaces

Motility assays and microtiter dish biofilm assays were performed as described previously by O'Toole et al. [28], [50].

### Initial attachment and biofilm formation on human airway epithelial cells

Attachment and biofilm formation on epithelial cells by *P. aeruginosa* PAO1 WT and Δ*pcs* mutant was examined using a protocol previously described by Anderson and O'Toole with slight modifications [29], [30]. *P. aeruginosa* was grown on CFBE epithelial cells (CFBE41o^−^ human bronchial epithelial cells homozygous for the ΔF508 mutation of CFTR). Epithelial cells were seeded at a concentration of 2×10^5^ cells/well in 24-well tissue culture plates and maintained in minimal essential medium (MEM) (Mediatech, Herndon, VA) with 10% fetal bovine serum, 2 mM L-glutamine, 50 U/ml penicillin, and 50 µg/ml streptomycin. The cells were grown at 37°C and 5% CO_2_ for 7 to 10 days to allow the formation of confluent cell monolayers and tight junctions. *P. aeruginosa* was inoculated at a concentration of 1.2×10^7^ CFU/ml in 0.5 ml MEM/well (without fetal bovine serum, penicillin, or streptomycin). The plates were incubated at 37°C and 5% CO_2_. For enumerating the number of cells after the initial attachment phase (after 1 hour of co-culture) the epithelial cells were gently washed with phosphate-buffered saline (PBS) to remove planktonic bacteria, and the cells were treated with 0.1% Triton X-100 for 10 minutes to lyse the epithelial cells. The lysate was vortexed, serially diluted, and plated on LB and the CFUs/well were enumerated. To assess biofilm formation, the supernatant in the wells was replaced after 1 hour of initial inoculation with fresh MEM and the plates were incubated at 37°C and 5% CO_2_ for 5 hours. After 5 hours the epithelial cells were rinsed with PBS and treated with 0.1% Triton X-100 to lyse the epithelial cells. CFUs in the lysates were enumerated as mentioned above. Each assay was performed at least in triplicate.

### *P. aeruginosa –Candida albicans* co-culture experiments

Co-culture experiments and microscopy were performed as described by Hogan et al. previously [31]. *P. aeruginosa* and constitutively-filamentous *Candida albicans nrg1/nrg1* [51] strain were inoculated into MOPS-glucose medium with or without choline as described in the text, and co-cultured for 48 hours. Aliquots from co-culture tubes were observed under a phase contrast microscope to analyze biofilm formation on the fungal surface.

### T3SS and PlcH assays

While *P. aeruginosa* PAO1 strains can form biofilms on airway epithelial cells in a co-culture model, PA14 strains are highly cytotoxic and lyse the epithelial cells [29], [30]. The T3SS-mediated cytotoxicity of *P. aeruginosa* PA14 and its Δ*pcs* mutant derivative towards epithelial cells was examined using a protocol previously described by Anderson and O'Toole [29]. *P. aeruginosa* and CFBE epithelial cells were co-cultured for 5 hours, as described above. After the incubation period, the culture medium was collected from each well and centrifuged to sediment the bacteria. Cell death and cell lysis were quantified, based on the measurement of lactate dehydrogenase (LDH) activity released from the cytosol of damaged cells into the supernatant. LDH levels within the cell-free supernatant were assayed using the Promega CytoTox 96 nonradioactive cytotoxicity assay according to manufacturer's instruction (Promega, USA). Percent LDH release (marker of cell lysis) was calculated relative to that of the uninfected control, which was set at 0% LDH release, and that of cells lysed with Triton X-100, which was set at 100% LDH release. Phospholipase C activity was measured using p-nitrophenyl phosphorylcholine (NPPC) as described before by Kurioka and Matsuda [40]. Bacteria were grown overnight in 5 ml of MOPS medium with 20 mM Pyruvate with or without 5 mM choline at 37°C. The reaction buffer was 100 mM Tris-HCl (pH 7.2), 25% glycerol, and 20 mM NPPC. NPPC hydrolysis was detected by measuring the absorbance at 410 nm. Assays were performed in triplicate.

### Mouse Lung Infection

The *P. aeruginosa* mouse model of acute pneumonia was performed as previously described [41]. Briefly, overnight LB cultures of *P. aeruginosa* were measured by OD_600_, pelleted, washed twice with PBS, and resuspended to give ∼3×10^7^ *P. aeruginosa* cells in 40 µL. Actual inoculum was determined by serial dilution of the input bacterial suspension on Pseudomonas Isolation Agar (PIA, Difco). Adult male C57Bl/6J mice, 8–12 weeks old (Jackson Labs), were inoculated with ∼3×10^7^ CFU of *P. aeruginosa* PAO1 or isogenic Δ*pcs* via oropharyngeal aspiration following brief anesthesia with isoflourane. The mice were anesthetized 24 hours post-infection with intraperitoneal sodium pentobarbital, tracheas were cannulated and bronchoalveolar lavage fluid (BALF) collected. Lungs were excised and immediately placed into 1 mL of cold PBS followed by homogenization. Viable bacterial counts in lungs were determined by plating serial dilutions of organ homogenate onto PIA plates followed by incubation at 37°C for 24 hours. WBC counts in the BALF were done using an Advia automated cell counter (Siemens).

### Phenotypic characterization of *P. aeruginosa* PA14 Δ*pcs* using Biolog phenotypic microarrays

For global phenotypic characterization of the role of phosphatidylcholine in *P. aeruginosa*, Biolog microarrays were utilized [52]. *P. aeruginosa* PA14 WT and the isogenic Δ*pcs* mutant were tested for phenotypic changes using microtiter plates PM9–PM20. Plates PM9–PM20 measure sensitivities to high osmolality, pH and different classes of antibiotics, antimetabolites and other inhibitors (www.biolog.com). The assays were performed with IF-10 medium (Biolog, Hayward, CA) supplemented with 1 mM choline chloride. Under these conditions, *P. aeruginosa* forms PC in its membranes (analysis of phospholipid profiles by TLC, data not shown). Growth kinetics of PA14 WT and PA14 Δ*pcs* mutant were compared by monitoring the cell respiration under individual conditions of the array, based on reduction of a reporter tetrazolium dye. Incubation, recording and analysis of the phenotypic data was performed by Biolog (Hayward, CA) using the Omnilog® system.

### Statistical analyses

One-factor analysis of variance (ANOVA) and *t* tests were performed using Prism 5.0 (GraphPad Software).

## Supporting Information

Figure S1 **Phospholipid profiles of clinical isolates of** ***P. aeruginosa*** **grown overnight in LB medium.** Separation of phospholipids by 1-D thin layer chromatography and detection by charring with sulphuric acid solution. The labels NM1, NM2, M1 and M2 represent PL profiles of four independent clinical isolates. Spots were determined by comparison with migration of phospholipid standards run in parallel (data not shown). Figure is a representative image of observations from two independent experiments.
(DOCX)

Figure S2 **Levels of infiltrating white blood cells in bronchoalveolar lavage fluid (BALF) were similar in PAO1 WT and PAO1 Δ** ***pcs*** **mutant-infected mice.** WBC infiltration into the BALF as measured by automated counter (Advia). Mean ± SEM plotted for 6 mice/group and were not significantly different (P value >0.05).
(DOCX)

Figure S3 **Data for Biolog phenotypic microarray PM 9–20 comparing** ***P. aeruginosa*** **PA14 WT and PA14 Δ** ***pcs*** **mutant.** Sensitivity towards osmolytes (PM9), pH (PM10), antibiotics, antimicrobial peptides and chemical inhibitors (PM11-20) was tested in this study (1152 conditions were tested, the description of the plates are available on http://www.biolog.com/pdf/PM11-PM20.pdf). The growth kinetics of *P. aeruginosa* strains grown under different conditions for 24 hours were analyzed by Omnilog® system which monitored reduction of a tetrazolium dye due to bacterial respiration. In the figures, growth advantage of PA14 wild type is indicated as red, while that of the PA14 Δ*pcs* mutant is shown as green. When the strains grew equally well, the red and green kinetic curves overlapped which are displayed as yellow curves. Black boxes around individual wells indicate instances where differences in growth kinetics were observed. Two replicate runs were performed. While both runs showed some differences between the PA14 wild type and the PA14 Δ*pcs* mutant, it is important to note that most of these differences were not observed in the technical replicates. The phenotypes detected in the run shown include the wild type having a growth advantage in (PM10-G11) pH 9.5+TMAO, (PM15-F8) oleandomycin, (PM18-G2) triclosan, (PM18-H8) 2 phenyl-phenol, (PM19-G1) laurylsulfobetaine, (PM20-G12) 8-hydroxyquinoline, and the *pcs* mutant with an advantage in (PM12-F8) sulphathiazole, (PM16-C4) dicholorofuramide, (PM16-C9,10) cetylpyridinium chloride, (PM17-H2) cefsulodin, (PM20-D12) phenylmethylsulfonylfluoride, (PM20-F8) oxytetracycline, (PM20-H12) troleandomycin. In the run not shown, the wild type had a slight growth advantage in (PM9-G2) sodium phosphate pH 7.0 (50 mM), (PM11-C8) colistin, (PM12-E8) sulfadiazine, (PM15-D5) domiphen bromide, (PM16-E11) rifamycin, (PM16-F6,7) sodium selenite, (PM16-G3) chromium chloride, (PM19-F4) phenithicillin, (PM20-E12) hexachlorophene, and the Δ*pcs* mutant showed growth advantages in (PM12-D9,10) novobiocin, (PM15-E1,2) alexidine, (PM16-A9,10,11,12) 5-chloro-7-iodo-8-hydroxyquinoline, (PM16-F1) potassium tellurite. None of these results were observed in both of the replicate runs. In the run shown, PA14 WT had a growth advantage over PA14 Δ*pcs* in the presence of sodium lactate (PM9-F1,2) and 5-fluoroorotic acid (PM12-F9,10,11,12), and neither PA14 WT nor PA14 Δ*pcs* grew in the presence of the compounds in the replicate run. The growth of *P. aeruginosa* PA14 WT, Δ*pcs* mutant and Δ*pcs* complemented strain in media with either 5-fluroorotic acid and sodium lactate was tested. No differences were observed in the growth rates of PA14 Δ*pcs* when compared to the wild type strains (data not shown).
(DOCX)

Table S1 **Clinical isolates of** ***P. aeruginosa*** **synthesize PC but only during exogenous availability of choline.** The strains were grown overnight in LB, MOPS medium with 20 mM glucose and MOPS medium with 20 mM choline media. Phospholipids were extracted and the profiles were analyzed by thin-layer chromatography and charring as described. ‘+’ sign indicates synthesis of PC whereas ‘−’ sign indicates absence of PC synthesis. PC standards and phospholipids extracted from LB grown PAO1 and PA14 WT strains served as controls.
(DOCX)
