# Supplementary figures and images for: Absence of Membrane Phosphatidylcholine Does Not Affect Virulence and Stress Tolerance Phenotypes in the Opportunistic Pathogen Pseudomonas aeruginosa
Source: PLoS One. 2012 Feb 17;7(2):e30829. doi: 10.1371/journal.pone.0030829 (PMC3281885; doi:10.1371/journal.pone.0030829)

**Fig. S1.**

**
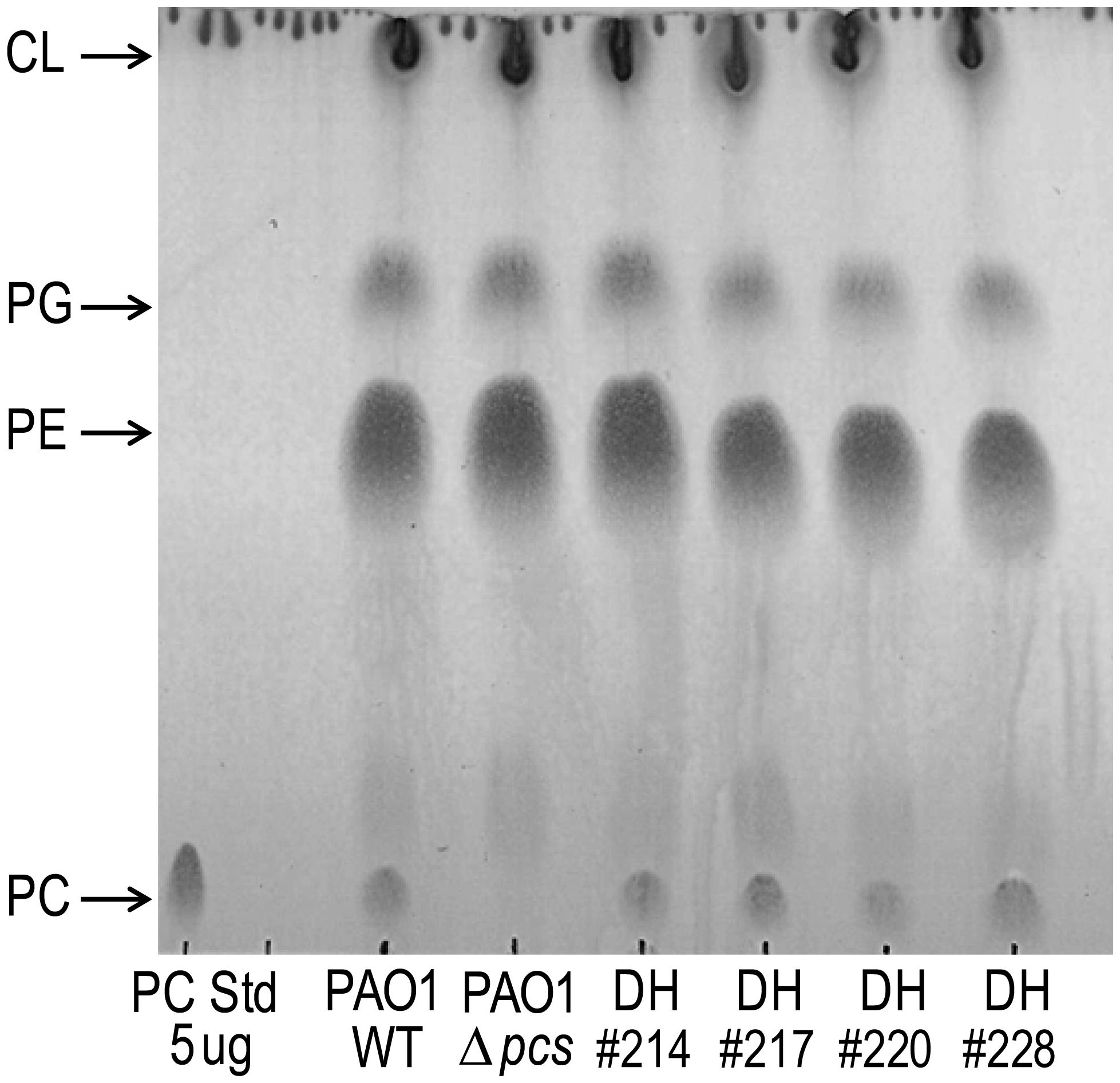
**

Supplement: Figure S1 — Phospholipid profiles of clinical isolates of P. aeruginosa grown overnight in LB medium. Separation of phospholipids by 1-D thin layer chromatography and detection by charring with sulphuric acid solution. The labels NM1, NM2, M1 and M2 represent PL profiles of four independent clinical isolates. Spots were determined by comparison with migration of phospholipid standards run in parallel (data not shown). Figure is a representative image of observations from two independent experiments. (DOCX) [file pone.0030829.s001.docx]

**Fig. S2.**


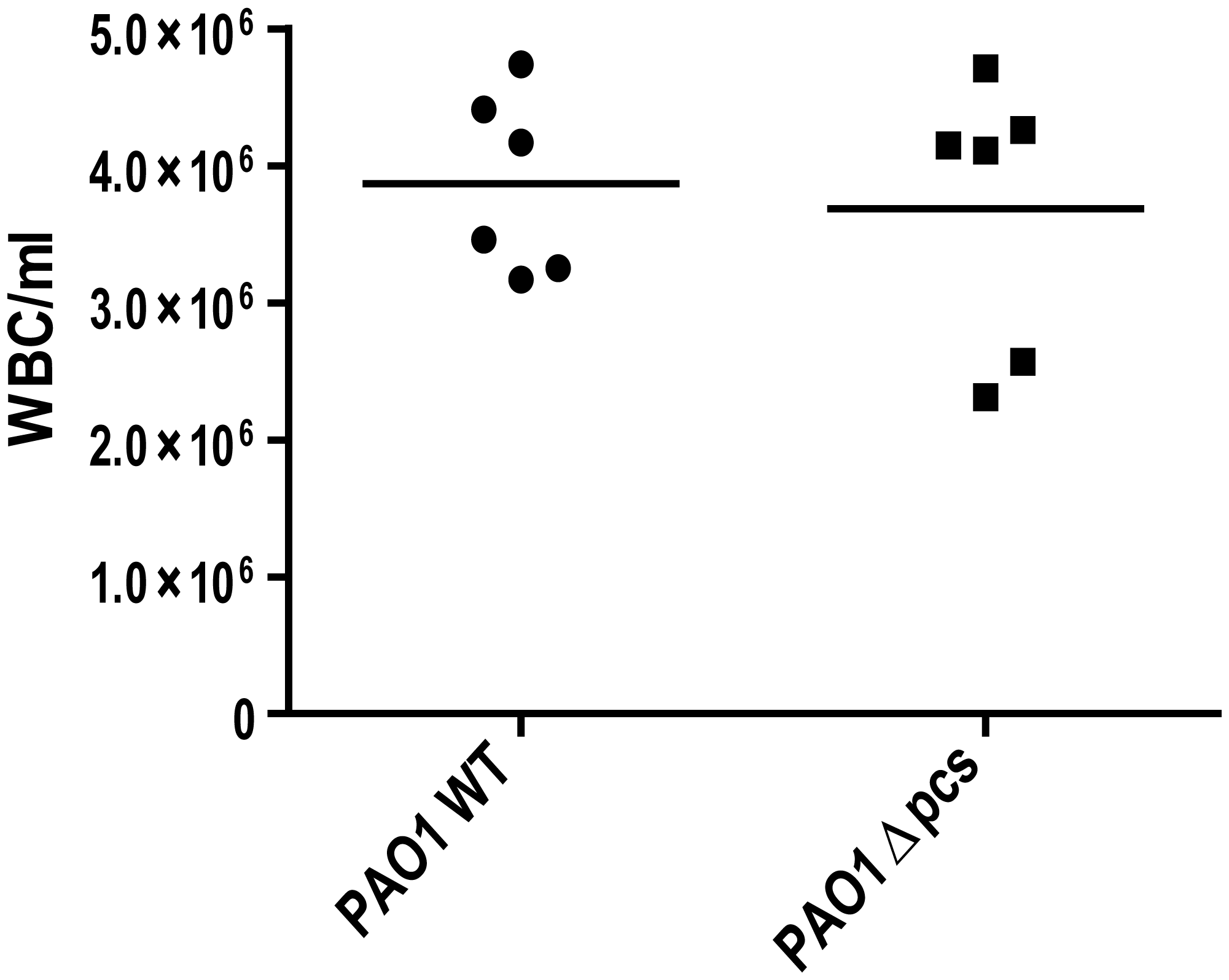

Supplement: Figure S2 — Levels of infiltrating white blood cells in bronchoalveolar lavage fluid (BALF) were similar in PAO1 WT and PAO1 Δ pcs mutant-infected mice. WBC infiltration into the BALF as measured by automated counter (Advia). Mean ± SEM plotted for 6 mice/group and were not significantly different (P value >0.05). (DOCX) [file pone.0030829.s002.docx]

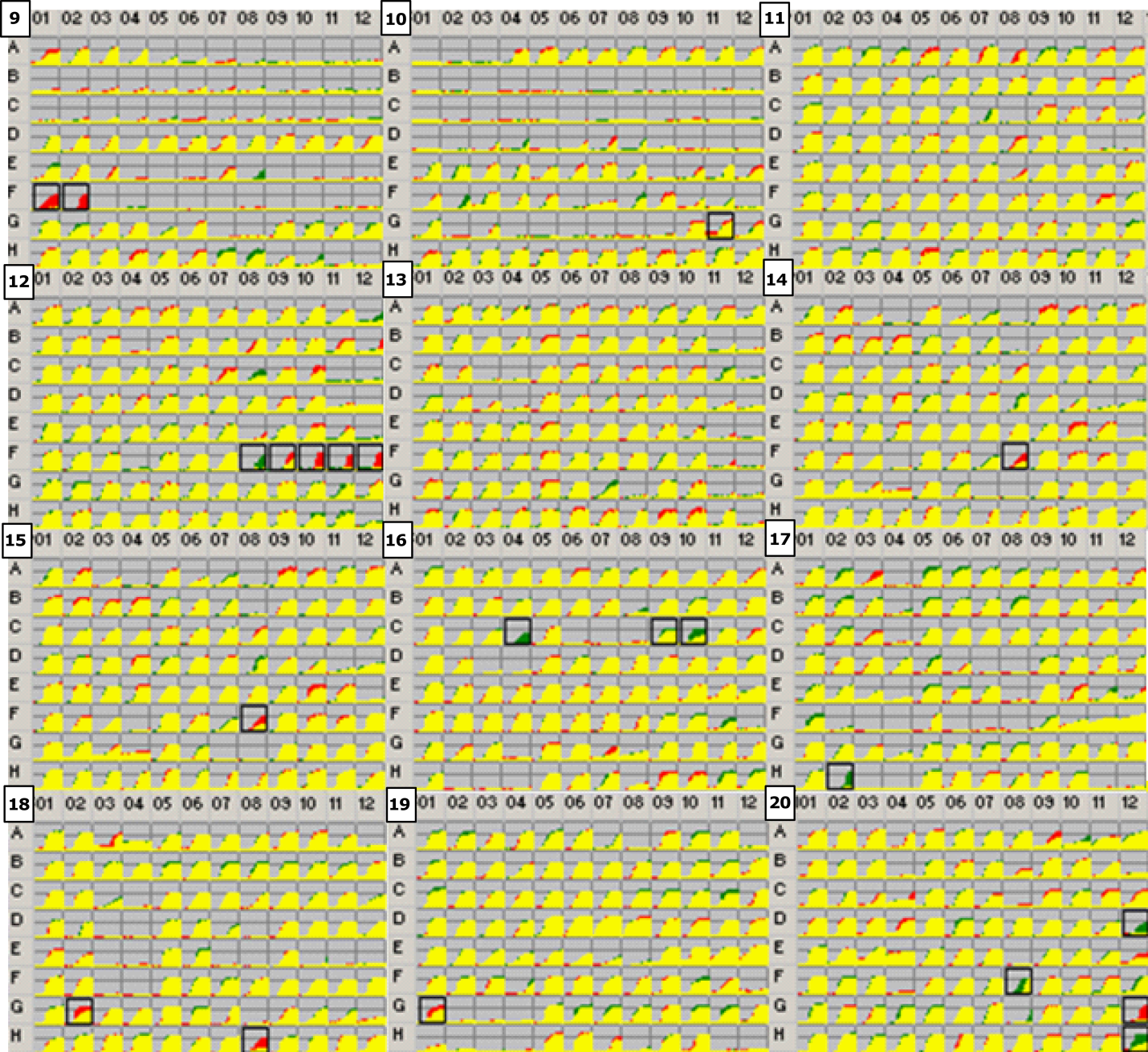
**Fig. S3.**

Supplement: Figure S3 — Data for Biolog phenotypic microarray PM 9–20 comparing P. aeruginosa PA14 WT and PA14 Δ pcs mutant. Sensitivity towards osmolytes (PM9), pH (PM10), antibiotics, antimicrobial peptides and chemical inhibitors (PM11-20) was tested in this study (1152 conditions were tested, the description of the plates are available on http://www.biolog.com/pdf/PM11-PM20.pdf). The growth kinetics of P. aeruginosa strains grown under different conditions for 24 hours were analyzed by Omnilog® system which monitored reduction of a tetrazolium dye due to bacterial respiration. In the figures, growth advantage of PA14 wild type is indicated as red, while that of the PA14 Δpcs mutant is shown as green. When the strains grew equally well, the red and green kinetic curves overlapped which are displayed as yellow curves. Black boxes around individual wells indicate instances where differences in growth kinetics were observed. Two replicate runs were performed. While both runs showed some differences between the PA14 wild type and the PA14 Δpcs mutant, it is important to note that most of these differences were not observed in the technical replicates. The phenotypes detected in the run shown include the wild type having a growth advantage in (PM10-G11) pH 9.5+TMAO, (PM15-F8) oleandomycin, (PM18-G2) triclosan, (PM18-H8) 2 phenyl-phenol, (PM19-G1) laurylsulfobetaine, (PM20-G12) 8-hydroxyquinoline, and the pcs mutant with an advantage in (PM12-F8) sulphathiazole, (PM16-C4) dicholorofuramide, (PM16-C9,10) cetylpyridinium chloride, (PM17-H2) cefsulodin, (PM20-D12) phenylmethylsulfonylfluoride, (PM20-F8) oxytetracycline, (PM20-H12) troleandomycin. In the run not shown, the wild type had a slight growth advantage in (PM9-G2) sodium phosphate pH 7.0 (50 mM), (PM11-C8) colistin, (PM12-E8) sulfadiazine, (PM15-D5) domiphen bromide, (PM16-E11) rifamycin, (PM16-F6,7) sodium selenite, (PM16-G3) chromium chloride, (PM19-F4) phenithicillin, (PM20-E12) hexachlorophene, and the Δpcs mutant showed growth adva [file pone.0030829.s003.docx]
