# Supplementary material for: Absence of Membrane Phosphatidylcholine Does Not Affect Virulence and Stress Tolerance Phenotypes in the Opportunistic Pathogen Pseudomonas aeruginosa
Source: PLoS One. 2012 Feb 17;7(2):e30829. doi: 10.1371/journal.pone.0030829 (PMC3281885; doi:10.1371/journal.pone.0030829)
Supplement: Table S1 — Clinical isolates of P. aeruginosa synthesize PC but only during exogenous availability of choline. The strains were grown overnight in LB, MOPS medium with 20 mM glucose and MOPS medium with 20 mM choline media. Phospholipids were extracted and the profiles were analyzed by thin-layer chromatography and charring as described. ‘+’ sign indicates synthesis of PC whereas ‘−’ sign indicates absence of PC synthesis. PC standards and phospholipids extracted from LB grown PAO1 and PA14 WT strains served as controls. (DOCX) [file pone.0030829.s004.docx]

**Table S1. Clinical isolates of *P. aeruginosa* synthesize PC but only during exogenous availability of choline.** The strains were grown overnight in LB, MOPS medium with 20mM glucose and MOPS medium with 20mM choline media. Phospholipids were extracted and the profiles were analyzed by thin-layer chromatography and charring as described. ‘+’ sign indicates synthesis of PC whereas ‘-’ sign indicates absence of PC synthesis. PC standards and phospholipids extracted from LB grown PAO1 and PA14 WT strains served as controls.

|  |  |  |  |  |  |  |  |  |
| --- | --- | --- | --- | --- | --- | --- | --- | --- |
|  |  |  | **Phosphatidylcholine synthesis** | | |  |  |  |
| **Internal isolate #** | **Source** | **Mucoidy** | **LB** | **MOPS 20mM Glu** | **MOPS 20mM Cho** |  |  |  |
| DH211 | a |  | + | - | + |  |  |  |
| DH212 | a |  | + | - | + |  |  |  |
| DH214 | b |  | + | - | + |  |  |  |
| DH215 | b |  | + | - | + |  |  |  |
| DH217 | a |  | + | - | + |  |  |  |
| DH220 | a | + | + | - | + |  |  |  |
| DH228 | a | + | + | - | + |  |  |  |
| DH1100 | c |  | + | - | + |  |  |  |
| DH1101 | c |  | + | - | + |  |  |  |
| DH1103 | c |  | + | - | + |  |  |  |
| DH1104 | c |  | + | - | + |  |  |  |
|  |  |  |  |  |  |  |  |  |
| ***Sources of clinical isolates*** | | |  |  |  |  |  |  |
| a. Dr. Jane Burns, Dept. of Pediatrics, Seattle Children's Hospital, WA, USA | | | | | | |  |  |
| b. Dr. Joseph Schwartzman, Dept. of Pathology, Dartmouth Medical Center, NH, USA | | | | | | | | |
| c. Ms. Deborah Henry, Dept of Pediatrics, B.C. Research Institute, BC, Canada | | | | | | |  |  |
